# Pain catastrophizing, pain sensitivity and fear of pain are associated with early life environmental unpredictability: a path model approach

**DOI:** 10.1186/s40359-022-00800-0

**Published:** 2022-04-10

**Authors:** Eszter Simon, András N. Zsidó, Béla Birkás, Árpád Csathó

**Affiliations:** 1 Department of Behavioral Sciences, Medical School, University of Pécs, Szigeti str. 12, 7624 Pécs, Hungary; 2 Institute of Psychology, University of Pécs, Pécs, Ifjúság str. 6, 7624 Pécs, Hungary

**Keywords:** Pain sensitivity, Pain catastrophizing, Fear of pain, Environmental unpredictability, Early life experiences

## Abstract

**Background:**

Socioeconomic disadvantages in the childhood environment might strongly influence beliefs and behavior characterizing the adult years. When children experience unpredictable and adverse situations, they develop an unpredictability schema with the core belief that situations are unpredictable.

**Methods:**

In two studies, we examined the association of childhood socioeconomic disadvantages with self-reported pain sensitivity, pain catastrophizing, and pain-related fear. Multidimensional survey measures were used to assess environmental conditions experienced in childhood. In addition, participants completed the Pain Catastrophizing Scale, Pain Sensitivity Questionnaire, Body Awareness Questionnaire, Unpredictability Schema Questionnaire, and Fear of Pain Questionnaire. In Study 1 (N = 252), in separate models, we examined pain sensitivity and pain catastrophizing of a community sample of pain-free young individuals in association with their childhood experiences. In Study 2 (N = 293), in a new sample, but with a wider age range, we examined the association of early life socioeconomic disadvantages with pain-related fear. In both studies, the predictions were tested with Structural Equation Modeling. Our models constituted a path from childhood socioeconomic status and household unpredictability to pain variables via the factors of family resources, unpredictability schemas, and body awareness.

**Results and conclusions:**

The findings converged on the conclusion that individuals experiencing disadvantageous early life conditions tended to have an elevated level of pain catastrophizing, higher perceived sensitivity to pain, and higher level of pain-related fear. These associations were mediated by an unpredictability schema and body awareness.

**Supplementary Information:**

The online version contains supplementary material available at 10.1186/s40359-022-00800-0.

## Background

Beyond the sensory determinants of pain, many aspects of personality, affection, and the socioeconomic environment, as well as the diverse interactions of these aspects, have been observed to have a marked influence on individual pain experience and ability to cope with pain [1, 2]. Of the adverse socioeconomic aspects, living in poverty, social marginality, and poorer educational background have been identified as potential risk factors for elevated pain states, both in chronic and acute conditions [3]. In line with these previous observations, the aims of the present survey-based studies were to contribute to the understanding of how the different aspects of pain perception are potentially altered by disadvantageous socioeconomic conditions and unpredictability experienced in childhood. In Study 1, we tested the association of pain sensitivity and pain catastrophizing with childhood environmental conditions via the mediation of perceived environmental unpredictability and body awareness. In Study 2, we examined the associations of fear of pain with the same childhood environmental conditions and via the same mediators as those in Study 1.

The objectives aimed were based on previous studies widely observing associations of socioeconomic disadvantages with the pain experience in clinical and non-clinical populations [3–5]. For example, in a large sample, Dorner et al. [5] observed a negative linear association between socioeconomic status and pain-related experiences, including the prevalence of severe pain, number of indicated painful body sites, intensity of pain and greater disability through pain. The latter factor, reported disability through pain, was found to be greater in those with lower socioeconomic status, even after controlling for somatic and psychological impairments. Similarly, high socioeconomic disadvantage was confirmed to be associated with many specific clinical conditions, such as orofacial pain [4], chronic widespread body pain [6, 7], and neuropathic pain [8].

Importantly, reports of pain in adulthood were associated not only with actual socioeconomic circumstances but also with those experienced in childhood [9, 10]. A 45-year-long follow-up study demonstrated that childhood social class might have an inverse relationship with the magnitude of most regional pain and chronic widespread pain [9]. Not all studies, however, support this conclusion. For example, some studies on low back pain found unclear or weak indication of the relationship between social factors in childhood and the prevalence of pain symptoms in adulthood [11, 12]. These diverse conclusions suggest that the processes linking childhood environment with adult pain behavior might be derived from many environmental causes and act via complex mechanisms. Based on a model addressing the associations of childhood socioeconomic status with adult health outcomes [12], childhood environment may impact on pain via many detrimental effects on the maturation of psychological and physiological mechanisms. In particular, growing up with socioeconomic disadvantages (e.g., with low housing quality, high crowding, toxic exposure, and adverse social climate) increases the likelihood that children will encounter adverse physical and psychosocial conditions, resulting in impaired emotional regulation, stress control, and cognitive functions [12]. In line with this model, it has been found that children who were exposed to physical and social adversities experienced an increased risk of chronic widespread pain in adulthood of up to 50–100% [9]. Furthermore, childhood abuse, poor early life functioning, and stressful life events have also been associated with the occurrence of chronic pain syndrome [13] and pain sensitivity [14].

Importantly, socioeconomic conditions experienced in childhood are suggested to be markers for environmental stability or predictability [15–17]. A low-quality, low-resource environment offers children less stability and the possibility of a more unpredictable, chaotic day-to-day life. This concept about the association of socioeconomic disadvantages and environmental unpredictability is not identical to other psychological constructs describing distressing childhood circumstances, such as childhood maltreatment [18–20] and adverse childhood experiences [19, 21]. These constructs represent mainly the traumatic events, neglect, and physical and sexual abuses experienced in childhood [21]. Such traumatic experiences can, of course, contribute to children developing a view of an unsafe, uncertain environment, but the psychological constructs built upon these experiences and their operationalizations largely miss the consideration of socioeconomic circumstances and the fluctuations in environmental conditions across space and time [21, 22].

Most of the aforementioned studies emphasize the need for further research into the direction of the complex causality that appears to connect childhood environmental conditions with adult pain experience. Therefore, in the two studies reported here, we tested a pathway model inspired by the model published recently by Proffitt Leyva and Hill [23]. Their model assumes that the socioeconomic status in childhood generates an unpredictability schema via the socioeconomic and parental environment. The general character of the schema depends on the quality and consistency of the socioeconomic and parental environment experienced. If children are exposed to a consistent, stable, and advantageous environment then they develop a schema that facilitates a view that the environment and life situations are predictable and controllable. In contrast, in an inconsistent, disadvantageous environment, the schema of children will represent an unpredictable world where control over situations is hardly feasible [24]. This schema, then, becomes a working model of the world in predicting the controllability of life events and this might influence many facets of behavior, including self-regulatory strength, future planning [25], and other mental processes that contribute to body sensation [23]. In accordance with other studies suggesting an interaction between developmental stress and the perception of bodily processes [26–28], Proffitt Leyva and Hill [23] showed that a more developed unpredictability schema can be linked to lower body awareness. Body awareness is not a unitary construct; in short, it can be considered a recognition ability of and attentiveness to different body signals [29]. The self-report questionnaires operationalizing body awareness, such as those used in the current studies, measure how individuals perceive their own sensitivity to body signals, and also the extent to which they feel to be attended to their body signals [30]. Too high or low levels of body awareness can lead to inaccurate perception and misinterpretation of body functions associated with an inappropriate intensity of emotions [31]. A close link between body awareness and pain perception is also frequently suggested [32–34], arguing that improved body awareness can facilitate self-regulative pain processes [31]. Accordingly, promoting interoceptive processes, such as improving body awareness, might be a key factor in pain management [32, 35, 36].

Furthermore, it is plausible to assume that the unpredictability schema works as an important cognitive factor in transferring the effects of the childhood environment to pain behavior and pain-related attitudes. This assumption is in line with many studies showing that the perception of controllability is a critical aspect of pain-related neural activity and pain behavior [37–41]. Generally, pain events experienced with an enhanced feeling of uncontrollability have been found to be associated with higher pain intensity and more intense negative emotions [38, 39, 42].

In two studies, our overarching goal was to test the associations of self-perceived pain sensitivity, pain catastrophizing, and fear of pain with socioeconomic conditions and unpredictability via the mediation of body awareness. We predicted that individuals having a schema with a more developed sense of unpredictability would have an elevated level of pain catastrophizing, higher sensitivity to pain, and higher pain-related fear. In Study 1, pain sensitivity and pain catastrophizing were assessed and tested separately in a sample of young pain-free adults. Pain catastrophizing refers to the tendency to focus on and to amplify the negative emotional value of pain sensations and to feel helpless when dealing with pain [43]. Pain sensitivity refers to the subjective experience of pain intensity in different life situations [44]. Both pain catastrophizing and sensitivity have importance in behavioral responses to actual or anticipated harmful, painful events. We predicted that both catastrophizing and sensitivity could be enhanced with a higher extent of unpredictability emerging from socioeconomic disadvantages experienced in childhood (i.e., assessed by socioeconomic status, familial support, and household unpredictability) through the mediator role of body awareness.

In addition to being a major determinant of acute pain intensity, catastrophizing may also play an important role in the development and the maintenance of chronic pain [45, 46]. As one of the most influential pain models, the fear-avoidance model of pain [47–49] suggests catastrophizing attitude is the initial step toward the development of chronic pain. Pain catastrophizing facilitates an enhanced fear of pain and contributes to an avoidance behavior, which in turn results in disuse, depression, and disability leading to exacerbated pain experience (e.g., increased sensitivity to pain) [47–49]. Accumulating evidence supports the predictive value of this model in many chronic pain conditions [50]. Since fear of pain, the second important factor in the fear-avoidance model, was not included in the first study, we investigated this emotional aspect of pain in a separate second study. Specifically, in Study 2, we examined how the unpredictability schema rooted in socioeconomic disadvantages is related to fear of pain. We tested the association of the unpredictability schema with fear of pain, along with the same predictions as in Study 1. For exploratory reasons, in this study, we explored the predictions through a wider age range while controlling for depression.

## Study 1

### Methods

#### Participants

A total of 252 healthy individuals (females = 176) aged between 18 and 35 years (mean age: 24.6, SD = 4.72) participated in the study. The semPower function programmed in R [51, 52] indicated that a sample size of N_Model 1.1_ = 164 and N_Model 1.2_ = 160, respectively, yields a power of approximately 95% to reject a wrong model with an amount of misspecification corresponding to RMSEA (root mean square error of approximation) = 0.03 on alpha = 0.05 (df _Model 1.1_ = 1213, df _Model 1.2_ = 1159). Thus, the sample size of the study was sufficient to provide the appropriate statistical power.

The participants completed an anonymous online survey after providing informed consent. The survey was created and distributed using online survey administration software (Google Forms, Google), and the participants were recruited by online advertisements on Internet listservs and social media. Participants were unaware of the purposes and hypotheses of the study. They reported pain-free health conditions, that is, they had no current pain or history of any chronic illness associated with pain. The study was conducted in 2019, according to the principles of the Declaration of Helsinki, and was approved by the Ethical Committee of the University of Pécs Medical School.

#### Procedure and materials

We used multidimensional, retrospective measures to assess environmental conditions experienced in childhood. Specifically, with three items, we measured the family’s Socioeconomic status in childhood (e.g., “I grew up in a relatively wealthy neighborhood”; McDonald's ω = 0.77) [53]. Each item was rated on a 7-point scale (1: Strongly disagree, 7: Strongly agree), with a higher score indicating better socioeconomic conditions. Eight items with 5-point scales were used to assess familial support in material and non-material resources (1: Inadequate support, 5: Exceptional support, e.g., “familial support for food” and “parental attention”; McDonald's ω = 0.88) [54]. In addition, participants rated their childhood Household unpredictability using three items with 7-point scales (e.g., “Things were often chaotic in our house”; McDonald's ω = 0.78) [16]. One of the three items (“People often moved in and out of my house on a pretty random basis”) showed no variance in the responses and was, therefore, excluded from further analysis. Participants also completed the 7-item Unpredictability Schema Questionnaire to rate the perceived controllability of life events (e.g., “I can handle unexpected events,” “I give up easily,” and “I know what to expect from people in my life”; McDonald's ω = 0.63) [24]. Each item was rated on a dichotomous scale (0: True, 1: False). The body awareness of participants was assessed with the Body Awareness Questionnaire (BAQ-H; McDonald's ω = 0.83) [55]. The BAQ-H contains 17 items (e.g., “I notice differences in the way my body reacts to various foods” and “I can tell when I go to bed how well I will sleep that night”). Finally, pain catastrophizing was measured using the Pain Catastrophizing Scale (PCS), and pain sensitivity was measured using the Pain Sensitivity Questionnaire (PSQ). The 13-item PCS (McDonald's ω = 0.93) [56] measures pain perception by focusing on the different perspectives of catastrophizing: rumination (constant negative thoughts about pain), magnification (exaggeration of pain) and helplessness (feeling the inability to cope with pain). The 17-item PSQ (McDonald's ω = 0.93) measures the self-reported pain sensitivity to everyday painful situations of individuals [44, 57]. The objectives and design of Study 1 are summarized in Table 1.

**Table 1:** Objectives and design of Study 1 and Study 2

Study 1	Study 2
*Goal*: to test the associations of pain sensitivity, and pain catastrophizing with childhood environmental conditions and perceived unpredictability via the mediation of body awareness *Prediction*: both pain catastrophizing and pain sensitivity could be enhanced with a higher extent of unpredictability emerging from disadvantages experienced in childhood through the mediator role of body awareness	*Goal*: to test the association of fear of pain with childhood environmental conditions and perceived unpredictability via the mediation of body awareness *Prediction*: fear of pain could be enhanced with a higher extent of unpredictability emerging from disadvantages experienced in childhood through the mediator role of body awareness
*Participants*	*Participants*
N = 252 Community sample Pain-free individuals by self-report *Age*: 18 and 35 years (mean age: 24.6, SD = 4.72) *Sex*: 176 females (69.84%), 76 males	N = 293 Community sample (different to that tested in Study 1) Pain-free individuals by self-report *Age*: 18 and 72 years (mean age: 33.6, SD = 11.70) *Sex*: 243 females (82.94%), 50 males
*Variables*	*Variables*
Childhood socioeconomic status Childhood Household Unpredictability Childhood Family Resources Unpredictability Schema Questionnaire Body Awareness Questionnaire Pain Sensitivity Questionnaire Pain Catastrophizing Scale	Childhood socioeconomic status Childhood Household Unpredictability Childhood Family Resources Unpredictability Schema Questionnaire Body Awareness Questionnaire Short Beck Depression Inventory Fear of Pain Questionnaire
*Analyses*	*Analyses*
Structural Equation Modeling Path Modell 1: socioeconomic status and household unpredictability → family resources → unpredictability schema → body awareness → *pain sensitivity* (*see also *Fig. 1) Path Modell 2: socioeconomic status and household unpredictability → family resources → unpredictability schema → body awareness → *pain catastrophizing* (*see also *Fig. 2) The models were controlled for sex	Structural Equation Modeling Path Modell: socioeconomic status and household unpredictability → family resources → unpredictability schema → body awareness → *Fear of Pain* (*see also *Fig. 3) The model was controlled for depression and sex

#### Data analyses

The predictions were tested with Structural Equation Modeling. Two models were constructed, one for pain sensitivity (Model 1.1) and one for pain catastrophizing (Model 1.2). Each model constituted a path from childhood socioeconomic status and household unpredictability to one of the pain variables (i.e., pain catastrophizing or pain sensitivity) via the intermediate factors of family resources, unpredictability schema and body awareness. The factor of family resources combines the provision of financial resources and emotional investment by the parents; therefore, it served as a direct antecedent factor of the unpredictability schema. The available and allocated resources in childhood have been found to show strong associations with childhood socioeconomic status and household unpredictability [54, 82]. Therefore, these factors were assessed and entered into the model as potential predictors of family resources.

The models were also controlled for sex based on statistical considerations and previous studies showing sex differences both in pain sensitivity and pain catastrophizing [58–63]. The two sexes were found to be significantly different in each scale of catastrophizing: females scored higher than males on the rumination (t(250) = 2.86, *p* < 0.01), magnification (t(250) = 2.06, *p* < 0.05), and helplessness (t(250) = 2.78, *p* < 0.01) subscales of the PCS. For the PSQ, no significant sex difference was found, but for a better comparison of the two models, and to take the findings of previous studies into account [58–63], this model was also controlled for sex.

We performed Structural Equation Modeling using the JASP statistical software version 0.14.0.0 for Windows, utilizing the lavaan package for R to assess fit measures for our proposed models. We used the diagonally weighted least squares (DWLS) estimator. To evaluate model fit, we used the relative chi-square (χ^2^/df), comparative fit index (CFI), Tucker–Lewis index (TLI) and root mean square error of approximation (RMSEA). The cut offs for good model fit were relative chi-square < 3 [64], CFI and TLI values of 0.95 or greater [65] and RMSEA values of 0.08 or lower [66]. The dataset of Study 1 is available in a data repository (https://data.mendeley.com/datasets/zryzjs773m/draft?a=7fbb61d6-eaae-49f8-8f03-e0354975ac5c).

### Results

#### Model 1.1: pain sensitivity

**Fig. 1:**
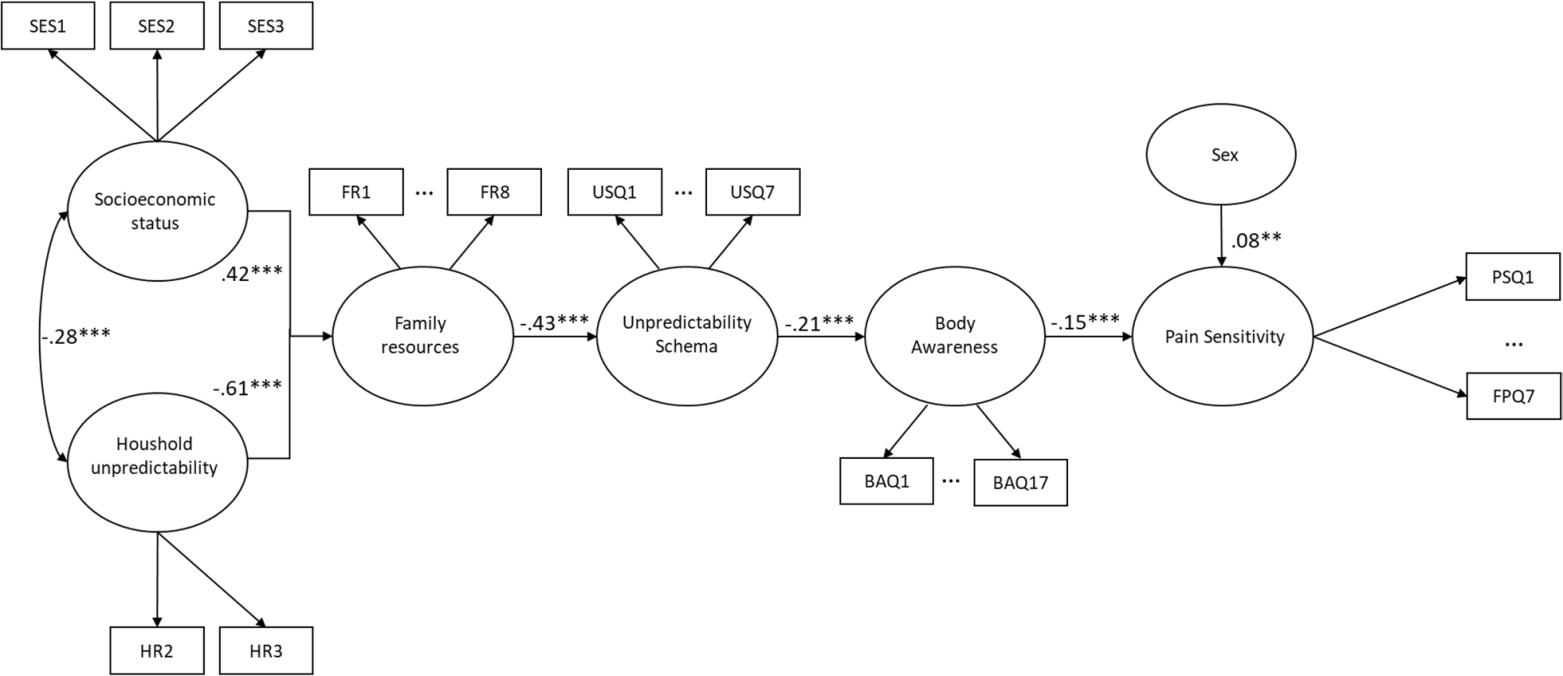
Model 1.1 in Study 1 for the path from childhood Socioeconomic status and Household unpredictability to Pain sensitivity (PSQ) via the intermediate factors of Family resources, Unpredictability schema and Body awareness. All reported estimates are the maximum likelihood standardized point-estimates (*Note*: *p < .05; **p < .01, ***p < .001)

The first model (Model 1.1) constituted a path from childhood socioeconomic status and household unpredictability to pain sensitivity via the factors of family resources, unpredictability schema and body awareness. Figure 1 depicts the model. The supplementary material presents the descriptive statistics (i.e., Additional file 1: Table S01) and the bivariate correlations between the model variables (i.e., Additional file 1: Table S02).

The test yielded a good model fit (χ^2^/df = 2.089, CFI = 0.958, TLI = 0.957, RMSEA = 0.066, 90%CI = [0.062–0.069]). In line with our hypothesis, we found that both Socioeconomic status (β = 0.42, *p* < 0.001) and Household unpredictability (β = − 0.61, *p* < 0.001) predicted Family resources. Family resources associated with Unpredictability schema (β = − 0.43, *p* < 0.001), which in turn associated with Body awareness (β = − 0.21, *p* < 0.001). Finally, Body awareness had a significant relationship with Pain sensitivity (β = − 0.15, *p* < 0.001) and Sex (β = 0.08, *p* < 0.01). After theoretical consideration, we allowed covariances between Socioeconomic status and Household unpredictability (β = − 0.28, *p* < 0.001). Furthermore, modification indices showed that allowing the residuals of BAQ2 and BAQ3 (MI = 46.65), BAQ1 and BAQ4 (MI = 44.73), BAQ14 and BAQ16 (MI = 40.63), FR1 and FR3 (MI = 35.16), and FR2 and FR3 (MI = 28.38) to correlate substantially improved model fit. Based on further theoretical (inspection of the content of the items) justification, we allowed the residuals of these items to correlate.

#### Model 1.2: pain catastrophizing

**Fig. 2:**
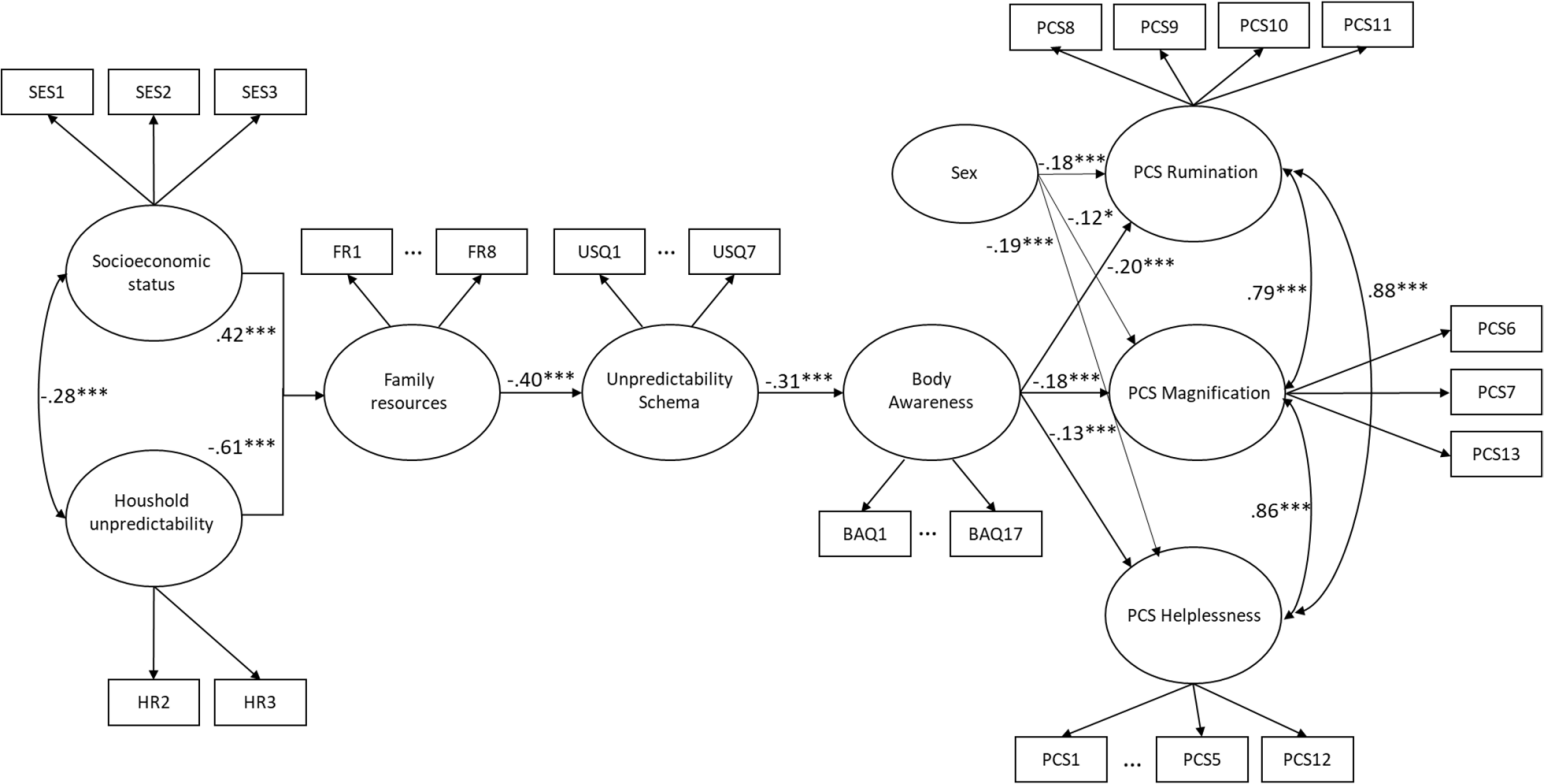
Model 1.2 in Study 1 for the path from childhood Socioeconomic status and Household unpredictability to Pain catastrophizing (PCS) via the intermediate factors of Family resources, Unpredictability schema and Body awareness. All reported estimates are the maximum likelihood standardized point-estimates (*Note*: *p < .05; **p < .01; ***p < .001)

The second model (Model 1.2) constituted a path from childhood socioeconomic status and household unpredictability to pain catastrophizing via the factors of family resources, unpredictability schema and body awareness. See Fig. 2 for the model, and the supplementary materials for descriptive statistics (i.e., Additional file 1: Table S01) and for the bivariate correlations between the model variables (i.e., Additional file 1: Table S02). The test, again, yielded a good model fit (χ^2^/df = 2.385, CFI = 0.952, TLI = 0.952, RMSEA = 0.074, 90%CI = [0.071–0.078]). Similar to Model 1.1, both Socioeconomic status (β = 0.42, *p* < 0.001) and Household unpredictability (β = − 0.60, p < 0.001) associated with Family resources. Then, Family resources associated with Unpredictability schema (β = − 0.40, *p* < 0.001), which associated with Body awareness (β = − 0.31, *p* < 0.001). Finally, Body awareness associated with all three PCS subscales: rumination (β = − 0.20, *p* < 0.001), Magnification (β = − 0.18, *p* < 0.001) and Helplessness (β = − 0.19, *p* < 0.001). Sex associated with all three PCS subscales: rumination (β = − 0.18, *p* < 0.001), Magnification (β = − 0.12, *p* < 0.05) and Helplessness (β = − 0.19, *p* < 0.001). Again, we allowed covariances between Socioeconomic status and Household unpredictability (β = − 0.28, *p* < 0.001): between PCS Rumination and PCS Magnification (β = 0.79, p < 0.001); between PCS Rumination and PCS Helplessness (β = 0.88, *p* < 0.001); and between PCS Magnification and PCS Helplessness (β = 0.86, *p* < 0.001). In addition, covariances were also allowed between the items (according to the modification indices) of BAQ2 and BAQ3 (MI = 48.33); BAQ1 and BAQ4 (MI = 46.18); BAQ14 and BAQ16 (MI = 38.71); FR1 and FR3 (MI = 36.04); and FR2 and FR3 (MI = 29.09).

### Discussion

Specifically, in Study 1, we tested the prediction that an uncertain, unpredictable childhood environment is related to the development of an unpredictability schema and that this is associated with an elevated level of pain catastrophizing and pain sensitivity through the mediator role of body awareness. The results of the analyses supported the prediction and converged on the conclusion that individuals experiencing disadvantageous early life conditions tend to have higher pain sensitivity and elevated levels of pain catastrophizing. The association between the early life conditions and the pain variables was mediated by an unpredictability schema and body awareness.

Of the two aspects of pain investigated in Study 1, the subjective sensitivity to pain seems to reflect mainly the sensory aspects of pain. There are indeed studies showing that self-reported sensitivity to hypothetical painful situations correlates with the actual physical experience of pain [44, 67], but nevertheless, there are also studies suggesting that subjective pain sensitivity is associated with the affective components of pain. For example, as in this study, studies have often observed positive associations of pain sensitivity with pain catastrophizing (e.g. [68]) and negative attitudes toward life events [57]. In addition, pain sensitivity measured by the PSQ has been found to have a negative correlation with resilience as a protective factor for pain vulnerability (e.g. [69]).

In Study 2, we further investigated the affective aspects of pain while examining pain-related fear (i.e., fear of pain). The model was controlled for the tendencies and symptoms of depression to reduce the influence of the non-pain specific affective attitudes (i.e., depression) on the results.

## Study 2

### Methods

#### Participants

A total of 293 individuals (females = 243) participated in Study 2 (in 2020). They were aged between 18 and 72 years (mean age = 33.6, SD = 11.70). The semPower function programmed in R [51, 52] indicated that a sample size of N = 147 yields a power of approximately 95% to reject a wrong model with an amount of misspecification corresponding to RMSEA = 0.03 on alpha = 0.05 (df = 1412). Thus, the sample size of the study was sufficient to provide the appropriate statistical power. Participants were unaware of the purposes and hypotheses of the study. Participants reported no current pain or history of chronic illness with pain. Like Study 1, participants completed an anonymous online survey and were recruited by online advertisements on Internet listservs and social media. The study was conducted according to the principles of the Declaration of Helsinki and was approved by the Ethical Committee of the University of Pécs Medical School.

#### Procedure and materials

In Study 2, participants responded to the same multidimensional measures about their childhood conditions as in Study 1 (for a comparison of Study 1 and 2, see Table 1). That is, they completed the measures of childhood Socioeconomic status (McDonald's ω = 0.82) [53], Household unpredictability (McDonald's ω = 0.66) [16] and Family resources (McDonald's ω = 0.89) [54]. In addition, we again assessed the cognitive schema of participants associated with unpredictability and uncontrollability with the Unpredictability Schema Questionnaire (McDonald's ω = 0.67) [24]. Like in Study 1, body awareness was assessed with the Body Awareness Questionnaire (BAQ-H; McDonald's ω = 0.84) [55].

Regarding the affective aspects of pain, in Study 2, participants answered the 9-item Fear of Pain Questionnaire-9 (FPQ-9; McDonald's ω = 0.79). The FPQ-9 measures individuals’ fear of pain by focusing on the different types of pain-related fear: Fear of Severe Pain (e.g., “Falling down a flight of concrete stairs”), Fear of Minor Pain (e.g., “Gulping a hot drink before it has cooled”) and Fear of Medical Pain (e.g., “Receiving an injection in your hip/buttocks”) [70]. To screen for depressive tendencies, we used the 9-item short form of the Beck Depression Inventory (BDI; McDonald's ω = 0.83) [71].

#### Data analysis

**Fig. 3:**
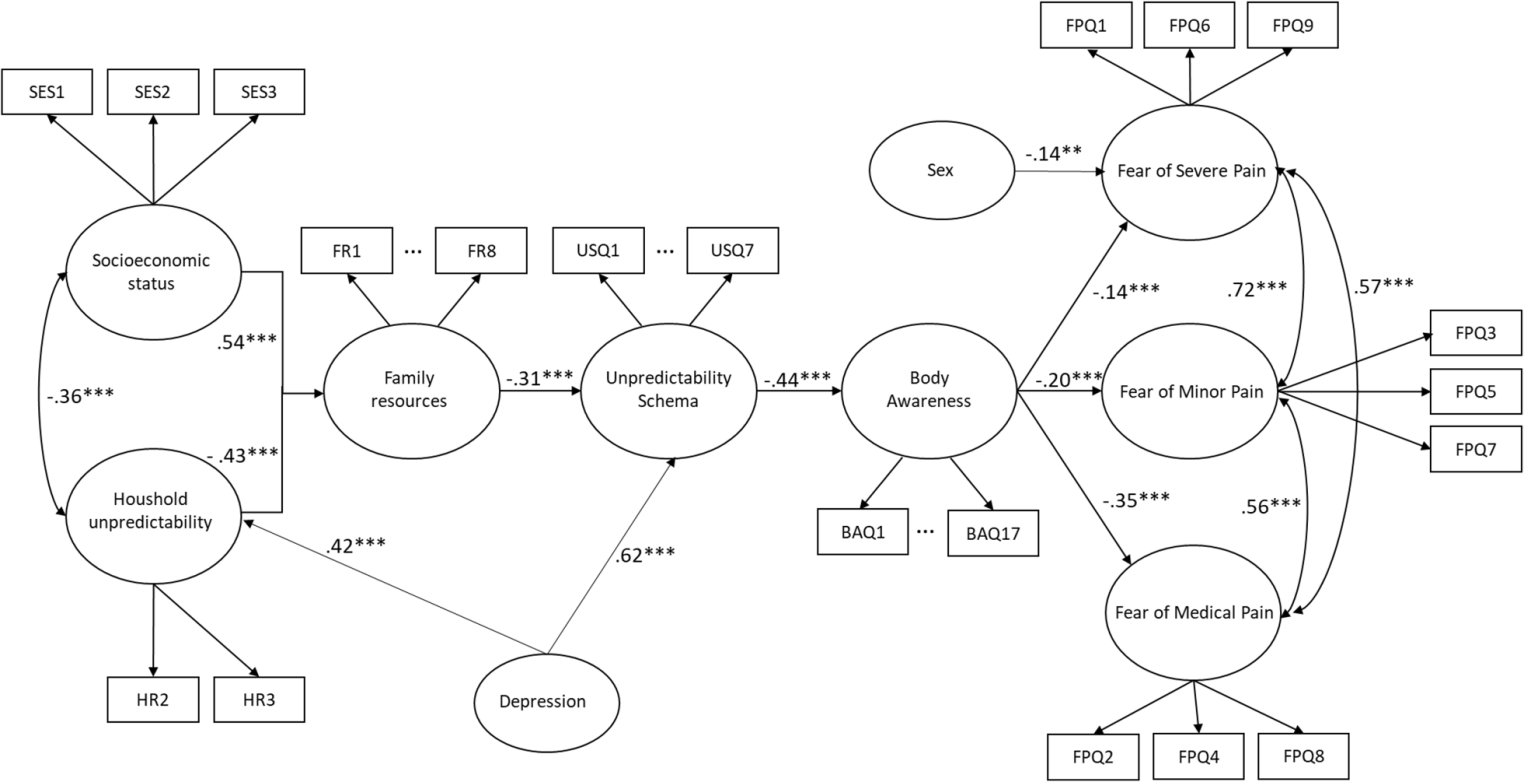
The model in Study 2 for the path from childhood Socioeconomic status and Household unpredictability to Fear of pain (FPQ-9) via the intermediate factors of Family resources, Unpredictability schema and Body awareness. All reported estimates are the maximum likelihood standardized point-estimates (MLE) (*Note*: *p < .05; **p < .01; ***p < .001)

The data analysis was identical to that described for Study 1. Specifically, the model constituted a path from childhood Socioeconomic status and Household unpredictability to Fear of pain via the factors of Family resources, Unpredictability schema and Body awareness. Based on Spearman correlation coefficients (*r* ≥ 0.25), Depression was used as a control for the Unpredictability Schema, Household unpredictability and Family resources. The age of the participants was not found to be correlated with any of the model variables with higher than or equal to the coefficient of 0.25; therefore, it was not entered as a control variable. Figure 3 shows the model. Although sexes were not found to be different in scores given for the FPQ-9, for comparisons with the models of Study 1, the SEM model tested in Study 2 was also controlled for sex. The dataset of Study 2 is also available in a data repository (https://data.mendeley.com/datasets/zryzjs773m/draft?a=7fbb61d6-eaae-49f8-8f03-e0354975ac5c).

### Results

The supplementary material presents the results of the descriptive statistics (Additional file 1: Table S03) and the bivariate correlations between the model variables (Additional file 1: Table S04).

The test yielded a good model fit (χ^2^/df = 1.315, CFI = 0.951, TLI = 0.950, RMSEA = 0.057, 90%CI = [0.054–0.060]). Both Socioeconomic status (β = 0.54, *p* < 0.001) and Household unpredictability (β = − 0.43, *p* < 0.001) associated with Family resources. Family resources was related to Unpredictability schema (β = − 0.31, *p* < 0.001), which then associated with Body awareness (β = − 0.44, *p* < 0.001). Finally, Body awareness showed a significant association with all three Fear of pain subscales: Fear of Severe Pain (β = − 0.14, *p* < 0.001), Fear of Minor Pain (β = − 0.20 *p* < 0.001) and Fear of Medical Pain (β = − 0.35, *p* < 0.001). Depression had a significant relation with Household unpredictability (β = 0.42, *p* < 0.001) and with the Unpredictability schema (β = 0.62, *p* < 0.001) but not with Household unpredictability (β = 0.01 *p* > 0.05). The model had similarly good fit without controlling for depression; the model indices without depression are shown in the supplementary material (S05). Sex associated only with the Fear of Severe Pain (β = − 0.14, *p* < 0.01). We allowed covariances between Socioeconomic status and Household unpredictability (β = − 0.36, *p* < 0.001); Fear of Severe Pain and Fear of Minor Pain (β = 0.72, *p* < 0.001); Fear of Severe Pain and Fear of Medical Pain (β = 0.57, *p* < 0.001); and Fear of Minor Pain and Fear of Medical Pain (β = 0.56, *p* < 0.001). Furthermore, modification indices showed that allowing the residuals of BAQ14 and BAQ16 (MI = 51.39), BAQ2 and BAQ3 (MI = 41.09), BAQ8 and BAQ9 (MI = 39.22), FR2 and FR3 (MI = 46.31) and FR4 and FR7 (MI = 44.13), to correlate substantially improved model fit. Based on further theoretical (inspection of the content of the items) justification, we allowed the residuals of these items to correlate.

### Discussion

In Study 2, with good model fits, we found evidence for our hypothesis that the general belief of individuals about an unpredictable, uncontrolled world (i.e., unpredictability schema) is associated with their pain-related fear. Specifically, it was evidenced that the unpredictability schema can be enhanced by the disadvantageous childhood conditions, and this schema is associated with fear of pain via the awareness to body signals. The findings of the model are independent from the effect of depression, suggesting that the model constituting paths from childhood environment to the aspects of fear of pain in adulthood is largely independent from the confounding effects of depression-related symptoms.

In the model of Study 2, fear of pain was found to be higher for individuals experiencing a more adverse childhood environment. Importantly, each subscale of the Fear of pain questionnaire fitted the model significantly, suggesting that, in addition to the overall negative reactions to pain (i.e., reactions to minor and severe pain), a specific aspect of pain-related fear (namely Fear of Medical Pain) may also be associated with adverse childhood conditions via the effect of the unpredictability schema.

## General discussion

The aim of the studies reported here was to evaluate the relevance of the unpredictability schema as a cognitive component in the process that connects the disadvantageous socioeconomic conditions with body awareness and certain aspects of pain perception (i.e., pain sensitivity, pain catastrophizing, and fear of pain). More specifically, we tested pathway models; each constituted a path from the childhood socioeconomic condition (assessed retrospectively) to pain sensitivity (Study 1), pain catastrophizing (Study 1), and fear of pain (Study 2), respectively, via the intermediate factors of family resources, unpredictability, and body awareness. The results from both studies support the conclusion that individuals experiencing unpredictable, disadvantageous early life conditions tend to have a more negative emotional appraisal of pain and a higher perceived sensitivity to pain in adulthood, and this association is mediated by the unpredictability schema and body awareness.

Chronically uncertain environments experienced in childhood are known to contribute to alterations in the physiological stress response system and to sensitize the brain to environmental stress in the long term [72]. More specifically, frequent exposure to adverse early life events can be manifested in a dysregulation of the hypothalamic–pituitary–adrenal (HPA) axis, the key biological cascade of the stress response [75, 76]. This stress-induced dysregulation seems to mainly appear in alterations of the negative feedback control of the HPA axis. The exact direction of changes in HPA functions, however, has not been fully identified yet, as previous empirical studies have observed both hypo- and hypersensitivity in the HPA system as a possible outcome of persistent early life stress [73–77]. In addition to altered HPA axis regulation, long-term neuromorphological changes have been observed in individuals being exposed to higher environmental stress during childhood. The amygdala, hippocampus, prefrontal cortical regions, and areas of the cingulate cortex were all found to be particularly vulnerable to adverse early life conditions [78–80]. Importantly, in the context of the current study, the stress-induced structural and functional changes may modify pain perception throughout diverse psychological mechanisms as an enhanced external focus of attention and the development of an unpredictability schema [14, 17, 23, 24]. Specifically, repeated and excessive activations of the stress-related neural systems and the structural–functional changes it entails may result in a vigilant and highly reactive stress response system. Such an enhanced reactivity to environmental stressors may come together with an externally driven attention focus [23, 28, 81]—individuals with a higher reactivity profile may be more focused on external environmental events (exteroceptive attention) along with a lessened focus on the identification of internal, body-related signals, such as pain (interoceptive attention) [17, 24, 32, 35]. In addition, these stress-induced neural alterations may also be interrelated with the development of the unpredictability schema characterized by the individual’s core belief that the outcomes of situations are unpredictable and uncontrollable [17, 23, 24, 82]. The unpredictability schema, on one hand, is a beneficial functional adaptation that is an important component of the coping repertoire that protects the individual from unexpected and adverse environmental effects. On the other hand, however, it might have undesirable psychosocial outcomes, mainly encouraging an avoidant behavioral style and weakening active attempts to solve problems and to regulate emotions [72]. The emotional appraisal of pain is usually benefitted by the ability to actively intervene in the painful situation and regulate pain-related emotions [83, 84]. Accordingly, a highly developed unpredictability schema counteracting with active problem-solving may lead to intensified and more adverse feelings relating to pain [85]. Our analyses support this association, showing that a more developed unpredictability schema is indirectly associated with increased pain sensitivity, pain catastrophizing, and fear of pain. As suggested by earlier studies, perceived control of life events might be crucial for pain therapies because the feeling of decreased control is associated with enhanced pain intensity and stronger negative emotions [37, 39, 42]. In line with this, our finding that lower levels of perceived control (represented by the unpredictability schema) are associated with elevated pain sensitivity and negative emotional states related to pain (i.e., catastrophizing and fear) suggests that improving the personal feeling of control may diminish the negative psychological attitudes toward pain. For example, stress inoculation training integrated with physiotherapy exercise has been found to be a successful treatment for reducing pain symptoms in patients with whiplash-associated disorders and symptoms of hyperarousal. The inoculation training included sessions aiming at improving the ability of patients to control stressful situations and coping strategies to manage their stress-related anxiety [86]. Similarly, there is much evidence that mindfulness meditation, cognitive therapy, and the combination of these two are highly effective treatments for chronic pain and act partly through mechanisms that improve perceived pain control [87–89].

In the models tested in the current study, body-related attentive processes (i.e., body awareness) directed the effect of the unpredictability schema to pain behavior. The finding that body awareness is linked both to the unpredictability schema and the sensitivity and affectivity to pain indicates the relevance of interventions that involve modulating the body awareness of individuals as a pain management tool [30, 34]. In other words, our findings can be considered as empirical support for recent body-based approaches that train patients to gain more control over their focus of attention and for improving interoceptive awareness as an important factor in pain behavior (see [90–92]). These therapies can be efficacious with numerous positive benefits, such as increased patient motivation to cope with pain and increased perceived control over pain, as well as decreased somatic complaints and tension [90–92].

Our analyses showed that a schema with a higher level of unpredictability associated with lower body awareness is negatively associated with each of the three aspects of pain catastrophizing (i.e. rumination, magnification and helplessness), sensitivity to pain, and fear of pain. The negative direction of the association between the unpredictability schema and body awareness is in line with earlier studies also mentioned above, suggesting that, in order to adapt to a harsh and unpredictable external environment, perception and attentional processing of external cues becomes more pronounced at the expense of interoceptive processes (see [28, 81]). This attentional shift may cause a decreased identification and awareness of pain signals [23].

Finally, it is important to note again that both pain catastrophizing and fear of pain are integrated affective components of the fear-avoidance model of chronic pain [93, 94]. Specifically, pain catastrophizing and fear of pain are two consecutive mediating factors in a cycle influencing perceived pain intensity and the chronic maintenance of pain. The current results suggest that both the core affective components (i.e., pain catastrophizing and fear of pain) of the model and the pain sensitivity they affect seem to be affected by the early life experiences of individuals. Further research might consider to investigate the effects of an adverse childhood environment specifically in the context of the fear-avoidance model. Notably, recent pain-stress models [95], however, suggest that acute and chronic pain may be differentially affected by stress. The relationship between acute pain and stress is best described by a linear trend; the relationship of chronic pain with stress, however, is more complex and seems to be rather curvilinear. That is, while low and high stress exposure can increase the risk of developing chronic pain, a moderate level of stress may protect individuals against the process of chronicity of pain sensation. This suggests that future empirical studies are particularly warranted to investigate precisely the extent to which the level of stress experienced in early life contributes to the perception of acute pain and the predisposition to chronic pain.

The present study has some limitations that should be considered while interpreting the results. First, the measures used to assess individual conditions experienced during childhood were retrospective and self-reported questionnaires. However, some studies suggest that these methods are positively associated with real observed early-life socioeconomic conditions [15]. Future studies should include different methods evaluating perceived unpredictability in order to gain a detailed causal insight into the elements of unpredictability schemas. Second, the community sample consisted of only young individuals (i.e., younger than 35 years of age) in Study 1 and predominantly young individuals in Study 2 where the age range was biased toward younger individuals, with only 34.5% of the sampling being over 35 years of age. This biased age distribution of the participants limits the generalizability of our findings and necessitates further studies including older participants. Third, an additional limitation of our study was that, although there is evidence [96–98] that females may have different pain sensitivity throughout the different phases of the menstrual cycle, our survey did not include any question referring to the actual phase of the menstrual cycle of female participants. Fourth, in both studies, many more female than male participants completed the surveys, which can also be considered a limitation of our studies. Fifth, many aspects of pain can be influenced by cultural factors [99, 100]. The fact that the present data were collected in a particular cultural and geographic population may undermine the generalizability of the results. Future studies may be needed to confirm the present findings among individuals from other cultural and geographical backgrounds. Sixth and finally, although the sample size in both studies was highly above the minimum requested by apriori power analyses, the sample of a few hundred participants might be considered modest and leave room for bias due to, for example, low population variability.

In summary, the results of both studies support previous findings concerning the positive association between childhood socioeconomic disadvantages and the pain of individuals [9, 12, 13]. The results suggest that a more developed unpredictability schema via a reduced level of body awareness may increase the perceived sensitivity to physical pain and intensify its affective and fear-related processing. Further investigations of the associations between stressful childhood experiences and body awareness and their effects on pain-associated factors are recommended for a more comprehensive understanding of the pain experience and further refinement of pain management methods.

## Supplementary Information

**Additional file 1**. Supplementary materials: descriptive statistics and bivariatecorrelations.

## Data Availability

The dataset is available in a data repository (see the link in the Methods section).

## Abbreviations

SES: Childhood socioeconomic status
FR: Family resources
HR: Household unpredictability
USQ: Unpredictability Schema Questionnaire
BAQ-H: Hungarian version of the Body Awareness Questionnaire
PCS: Pain Catastrophizing Scale
PSQ: Pain Sensitivity Questionnaire
FPQ-9: Fear of Pain Questionnaire 9 item version
BDI: Beck Depression Inventory
SEM: Structural equation modeling
DWLS: Diagonally weighted least squares estimator
χ^2^/df: Relative chi-square
CFI: Comparative fit index
TLI: Tucker-Lewis index
RMSEA: Root mean square error of approximation
MLE: Maximum likelihood standardized point-estimates
