# Supplementary material for: Pain catastrophizing, pain sensitivity and fear of pain are associated with early life environmental unpredictability: a path model approach
Source: BMC Psychol. 2022 Apr 10;10:97. doi: 10.1186/s40359-022-00800-0 (PMC8996610; doi:10.1186/s40359-022-00800-0)
Supplement: Supplementary file 1 — Additional file 1. Supplementary materials: descriptive statistics and bivariatecorrelations. [file 40359_2022_800_MOESM1_ESM.docx]

**Supplementary Materials**

**Pain catastrophizing, pain sensitivity and fear of pain are associated with early life environmental unpredictability: a path model approach**

**Table S01.** Descriptive statistics for the variables used in the models of Study 1.

| *Variables* | *Mean* | *SD* |
| --- | --- | --- |
| Socioeconomic status | 11.08 | 3.84 |
| Household unpredictability | 5.14 | 3.05 |
| Family resources | 31.49 | 6.11 |
| Body awareness | 4.68 | .90 |
| Unpredictability schema | 1.97 | 1.64 |
| Pain catastrophizing |  |  |
| - Rumination | 8.40 | 4.53 |
| - Magnification | 3.50 | 3.29 |
| - Helplessness | 8.05 | 5.73 |
| Pain sensitivity | 4.07 | 1.64 |

| *Variables* | 1. | 2. | 3. | 4. | 5. | 6. | 7. | 8. | 9. |
| --- | --- | --- | --- | --- | --- | --- | --- | --- | --- |
| 1. Socioeconomic status | — |  |  |  |  |  |  |  |  |
| 2. Household unpredictability | -.18** | — |  |  |  |  |  |  |  |
| 3. Family resources | .42*** | -.53*** | — |  |  |  |  |  |  |
| 4. Unpredictability Schema | -.10 | .27*** | -.28*** | — |  |  |  |  |  |
| 5. Body Awareness | -.01 | -.04 | .10 | -.26*** | — |  |  |  |  |
| 6. Pain Sensitivity | .07 | .15* | .02 | .11 | -.17** | — |  |  |  |
| 7. PCS Rumination | .02 | .23*** | -.07 | .29*** | -.15* | .34*** | — |  |  |
| 8. PCS Magnification | .11 | .21*** | -.03 | .21*** | -.13* | .35*** | .67*** | — |  |
| 9. PCS Helplessness | .07 | .24*** | -.05 | .25*** | -.09 | .34*** | .80*** | .69*** | — |

**Table S02.** Bivariate correlations (Spearman coefficients) between the variables used in the models of Study 1.

*Note*. **p* < .05. ***p* < .01. ****p* < .001; PCS: Pain Catastrophizing Scale

**Table S03.** Descriptive statistics for the variables used in the model of Study 2.

| *Variables* | *Mean* | *SD* |
| --- | --- | --- |
| Socioeconomic status | 10.94 | 4.15 |
| Household unpredictability | 5.16 | 3.05 |
| Family resources | 29.97 | 6.62 |
| Body awareness | 4.70 | .79 |
| Unpredictability schema | 1.84 | 1.70 |
| Fear of Pain |  |  |
| - Severe Pain | 9.24 | 3.00 |
| - Minor Pain | 5.52 | 2.36 |
| - Medical Pain | 7.48 | 3.18 |
| Depression | 14.42 | 4.40 |

| *Variables* | 1. | 2. | 3. | 4. | 5. | 6. | 7. | 8. | 9. | 10. |
| --- | --- | --- | --- | --- | --- | --- | --- | --- | --- | --- |
| 1. Age | — |  |  |  |  |  |  |  |  |  |
| 2. Depression | -.22^***^ | — |  |  |  |  |  |  |  |  |
| 3. Socioeconomic status | -.09 | -.21^***^ | — |  |  |  |  |  |  |  |
| 4. Household unpredictability | -.15^*^ | .35^***^ | -.32^***^ | — |  |  |  |  |  |  |
| 5. Family resources | -.18^**^ | -.25^***^ | .52^***^ | -.48^***^ | — |  |  |  |  |  |
| 6. Unpredictability schema | -.19^**^ | .48^***^ | -.25^***^ | .25^***^ | -.28^***^ | — |  |  |  |  |
| 7. Body awareness | .21^***^ | -.20^***^ | .11 | -.12^*^ | .14^*^ | -.31^***^ | — |  |  |  |
| 8. Fear of Severe pain | -.19^**^ | .14^*^ | -.07 | .08 | -.04 | .22^***^ | -.02 | — |  |  |
| 9. Fear of Minor pain | -.02 | .07 | -.04 | .12^*^ | -.08 | .10 | -.06 | .47^***^ | — |  |
| 10. Fear of Medical pain | -.24^***^ | .16^**^ | -.04 | .19^***^ | -.11 | .22^***^ | -.17^**^ | .42^***^ | .40^***^ | — |

**Table S04.** Bivariate correlations (Spearman coefficients) between the variables used in the model of Study 2.

*Note*. **p* < .05. ***p* < .01. ****p* < .001

**S05.** The FPQ model fits without controlling for depression.

The test yielded a good model fit (χ^2^/df = 2.133, CFI = .951, TLI = .950, RMSEA = .062, 90%CI = .059–.066]). Both Socioeconomic status (β = .52, *p* < .001) and Household unpredictability (β = -.46, *p* < .001) predicted Family resources. Family resources associated with Unpredictability schema (β = -.54, *p* < .001), which in turn associated with Body awareness (β = -.44, *p* < .001). Finally, Body awareness had a significant relationship with Fear of Severe Pain (β = -.10, *p* < .001), Fear of Minor Pain (β = -.18, *p* < .001) and Fear of Medical Pain (β = -.33, *p* < .001). Only Fear of Severe Pain had significant association with Sex (β = -.14, *p* < .05). After theoretical consideration, we allowed covariances between Socioeconomic status and Household unpredictability (β = -.45, *p* < .001) and the Fear of Pain subscales: Fear of Severe Pain and Fear of Minor Pain (β = .72, *p* < .001), Fear of Severe Pain and Fear of Medical Pain (β = .58, *p* < .001), Fear of Minor Pain and Fear of Medical Pain (β = .57, *p* < .001). Furthermore, modification indices showed that allowing the residuals of BAQ14 and BAQ16 (MI = 55.56), BAQ2 and BAQ3 (MI = 40.62), BAQ8 and BAQ9 (MI = 37.59), FR4 and FR7 (MI = 47.85), and FR2 and FR3 (MI = 45.73) to correlate substantially improved model fit. Based on further theoretical (inspection of the content of the items) justification, we allowed the residuals of these items to correlate.
